# Psychological outcomes in ethnically minoritised adolescents and young adults with cancer: A systematic review

**DOI:** 10.1177/13591045241301644

**Published:** 2024-12-17

**Authors:** Lynette WS Chan, Alan Hebben-Wadey, Chandrika Kambakara Gedara, James McParland

**Affiliations:** 1 Salomons Institute for Applied Psychology, 370981 Canterbury Christ Church University, UK; 2 8964 University College London Hospitals NHS Foundation Trust, UK

**Keywords:** Adolescents and young adults, cancer, psychological outcomes, ethnic minority, mental health, ethnically minoritised

## Abstract

**Background:**

Ethnic disparities in cancer prevalence and health outcomes have been widely documented in adults. However, less is known about the impact of ethnic differences in young cancer patients who present with complex needs along their developmental trajectories. The present review aimed to examine psychological outcomes amongst ethnically minoritised adolescents and young adults (AYAs) with cancer.

**Method:**

A systematic search was conducted on four databases using terms related to AYAs, cancer, ethnic minority and psychological outcomes. Quantitative studies of any design were included and screened against the eligibility criteria. Studies were rated for methodological quality and synthesised narratively.

**Results:**

Twelve studies conducted in the United States were identified with mostly moderate to low quality and the evidence was mixed. Six studies found ethnic disparities in psychological outcomes: the majority demonstrated that ethnically minoritised AYAs experienced significantly more distress compared to White peers with cancer. Hispanic youths were highlighted as a vulnerable group that fared worse in their mental health compared to other minoritised youths. Longitudinal data showed that minoritised AYAs experienced more marked improvement in their psychological health over time compared to Caucasians.

**Conclusion:**

There is emerging evidence regarding inter-ethnic differences in psychological outcomes amongst AYAs with cancer. However, the findings are inconsistent, reflecting methodological weaknesses and the complexities of intersectionality impacting on mental health. Further cross-cultural research is necessary to substantiate these findings and elucidate mechanisms behind these inequalities to promote more equitable healthcare.

## Plain language summary

People’s risks of getting cancer, how they manage and their likelihood to survive can vary across different ethnic backgrounds. However, we do not know whether these differences we see in adults also exist in younger cancer patients. To understand more about this younger group who may have additional needs as they grow up to become adults, we wanted to find out from existing research whether the mental health of young cancer patients from minoritised backgrounds differ compared to White young patients. We conducted an in-depth search on four databases and compared the results from the relevant research studies.

After comparing 12 studies, our results were:

• Half of the studies showed that minoritised young people struggle more with their mental health compared to White young people.
• Hispanic youths in particular had worse mental health amongst all groups.
• The studies measured different things and their results were mixed, so it is hard to compare or draw any firm conclusions about whether young cancer patients’ mental health differed between ethnic groups.

We need to do more research to confirm these results and to understand more about why some ethnic groups with cancer might have poorer mental health.

## Introduction

Cancer in adolescents and young adults (AYAs) has gained burgeoning attention over the last decade. The incidence rate of AYAs cancer has inflated markedly by 24% since the 1990s (Cancer Research UK, 2021). International oncology working groups have highlighted AYAs as a distinct population between the ages of 15–39 who exhibit substantial differences in the biology, aetiology and survivorship of cancer, compared to their younger or older counterparts (Ferrari et al., 2021). Whilst this categorisation may be helpful in refining oncology research, it encompasses a wide age range that reduces the heterogeneous experiences of a 15-year-old and a 39-year-old into a single population. This categorisation limits the extent of generalisability and comparison drawn from AYAs research across this diverse spectrum. Nevertheless, it represents a pivotal change in providing valuable insights to address AYAs’ age-specific concerns that were previously understudied.

Transitional aged AYAs are at a formative life stage of undergoing significant physiological and psychosocial development. Yet the disruptions that cancer bring to this developmental trajectory challenge the attainment of these important milestones. Many studies have reported elevated levels of psychological distress in AYAs with cancer compared to healthy peers (Sansom-Daly & Wakefield, 2013). Between 30% to 57% of AYAs with cancer reported clinical symptoms of depression, anxiety and post-traumatic stress (Kwak et al., 2013a; Lee et al., 2023). Whilst AYAs survival rate has improved significantly, AYAs survivorship comes along with its challenges as AYAs are at increased risk of experiencing long-term and late adverse effects, such as fertility challenges, cardiovascular conditions and secondary malignancies (Chao et al., 2016; Suh et al., 2020). AYA survivors are also less likely to engage in education or employment with decreased financial independence compared to AYAs without cancer, which are associated with distress and lowered self-efficacy (Janssen et al., 2022; Sisk et al., 2020). Over 80% of AYAs experienced clinically significant levels of fear about cancer recurrence (Shay et al., 2016), highlighting the detrimental psychological impacts that merit attention.

Epidemiological analysis on cancer survival rates in the United States (US) revealed that White AYAs have the highest survival rate of 89.1%, whilst non-Hispanic Black AYAs have the lowest despite having a 25% lower cancer incidence rate than the former (National Cancer Institute, 2022). The stark differences have prompted national inquiries into cancer inequalities (Marmot et al., 2020). A meta-analysis found that minoritised adult cancer patients, especially those identified as Hispanic, reported significantly worse psychological distress, depressive symptoms and quality of life than the White patients (Luckett et al., 2011). Meyer (2003) proposed the Minority Stress Model for understanding the disproportionate prevalence of mental health difficulties amongst minoritised groups. He postulated that individuals who experience a high degree of stigma and prejudice can develop chronic stress responses that lead to poor physical and mental health. The developmental and health predicament faced by AYAs with cancer, compounded with the challenges of identifying as ethnically minoritised are likely to exacerbate their distress.

To date, there is only one review examining psychological difficulties in ethnic minority adult cancer patients (Luckett et al., 2011), yet none have been conducted in the younger population. Therefore, a review of the literature pertaining to minoritised AYAs is timely and can illuminate on systemic racial disparities to drive actions narrowing the gap. This review aimed to synthesise and appraise the evidence base on psychological outcomes amongst ethnically minoritised AYAs with cancer and to investigate the extent of disparities in outcomes between different ethnic groups.

## Methodology

This review was completed in accordance with the Preferred Reporting Items for Systematic Reviews and Meta-analyses (PRISMA) guidelines and was published on PROSPERO (registration number: CRD42023466464).

### Search strategy

A systematic search was conducted on the eighth of October 2023 on PsycINFO, PubMed, Web of Science and CINAHL. Search terms used were grouped under ‘adolescents and young adults’, ‘cancer and oncology’, ‘ethnic or racial minority’ and ‘psychological outcomes’ (search strategy available in supplementary file 1). Reference lists of included studies and relevant reviews were searched for further eligible studies.

### Eligibility criteria

Studies were limited to those published in peer-reviewed journals in the English language after 2000. Quantitative studies of all designs were included. Participants were AYAs who had been diagnosed with cancer (any cancer type, stage or treatment status) between the ages of 15 and 39. Studies must include ethnicity analysis comparing any standardised measures of psychological distress, symptoms or emotional well-being outcomes in at least two ethnic groups. Studies were excluded if (i) they were case studies, case series or dissertations, (ii) only reported data in majority groups, or (iii) had a mean age of diagnosis under 15 to ensure that the review was not overshadowed by findings of paediatric cancer survivors.

### Study selection

**Figure 1.**
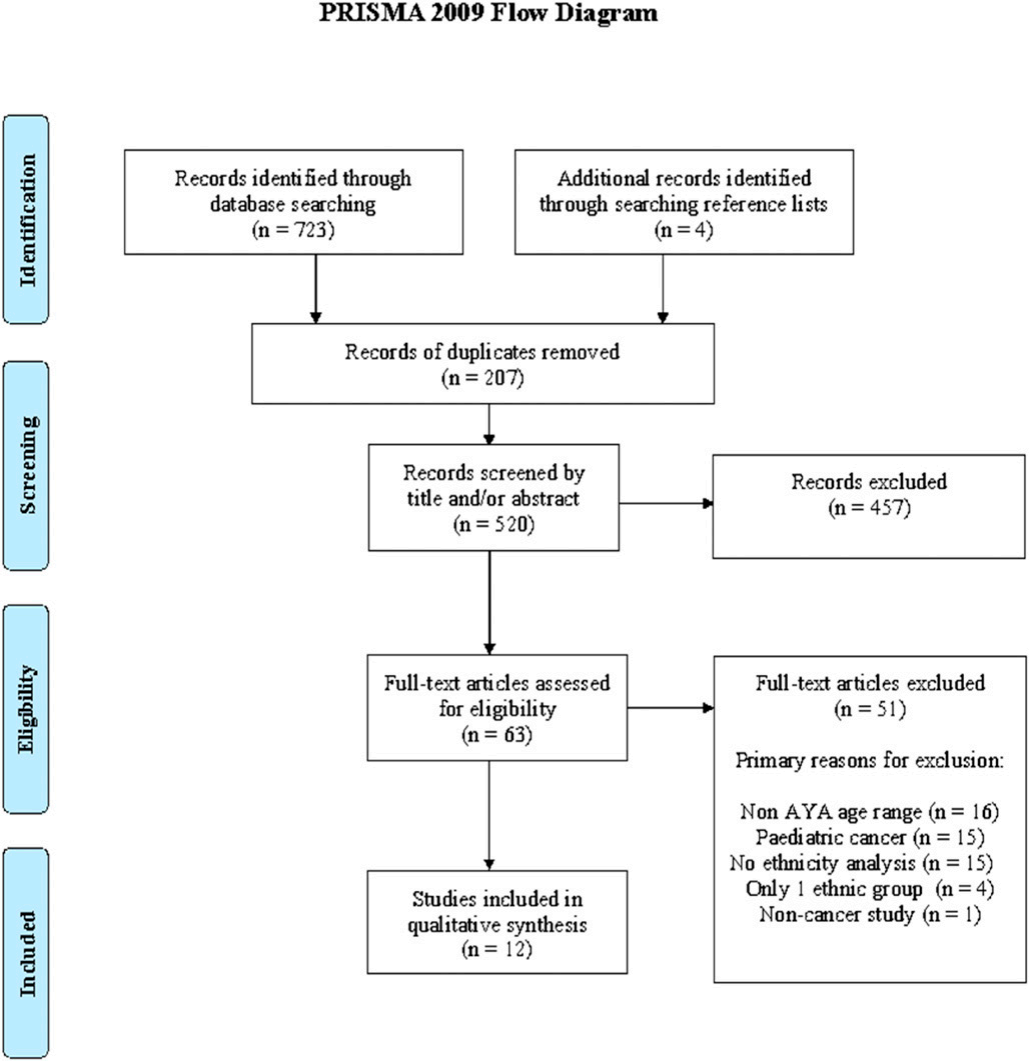
PRISMA flow diagram.

The search yielded 723 eligible studies and an additional four were identified through hand-searching reference lists of relevant studies. After screening by title and abstract, 63 studies were eligible for full-text review (Figure 1). Primary reasons for exclusions were participants not within the AYA age range and no ethnicity analysis.

### Data extraction

The Cochrane Data Extraction Tool was used as a guide to extract data from twelve included studies. Extracted information included: study methodology; participant demographics; recruitment; attrition rates and outcome measurement. Due to the heterogeneity in measuring ethnicity and outcomes across studies, data were aggregated descriptively for a narrative synthesis.

### Quality appraisal

Quality assessment of the studies was carried out using the Quality Assessment Tool for Observational Cohort and Cross-Sectional Studies developed by the National Heart, Lung and Blood Institute (2021). This tool was chosen for its capacity to assess methodological quality for a range of observational study designs. Each study was assigned a score for each criterion: ‘yes = 1 point’, ‘no = zero point’, ‘not reported = zero point’ or ‘not applicable = excluded from scoring’. These points were summed and calculated into a percentage that was converted into an overall quality score: below 33% equated to a low quality rating, 34%–66% corresponded to moderate and above 67% was considered as strong.

## Results

### Study characteristics

Twelve studies were identified. Four studies appeared to have overlapping datasets as evidenced by the same recruitment procedure through the same institutions during a matching period. Nevertheless, each study reported a different sample size, presented slight variations in demographics and examined diversified research questions and outcomes, hence, all were included in the review. The overall sample size was estimated to be 3886 participants (approximate pooled mean age of 27.9). This estimation has taken into account the four suspected duplicate datasets by only including the study with the largest sample size in the calculation to minimise over-inflation of participant count. More than 1623 participants identified as minoritised, constituting at least 42% of the full sample. All studies were conducted in the US and recruited participants with heterogeneous cancer types, disease stages and treatment status (Table 1).

**Table 1.** Summary of Included Studies.

Study	Setting	Design timepoints	Sample demographics *n* (% female), age range, mean age (SD), cancer type	Ethnic groups (*n*)	Psychological outcome measures	Main findings
Burgoyne et al. (2015)	USA, hospital-based	Retrospective cross-sectional study	*n* = 668 (58.7) Age = 18–39 31.1 (6.1) Numerous and heterogeneous cancer types, stages and treatment status	Caucasian (503) African American (101) Hispanic (31) Other (33)	Distress thermometer	**With Bonferroni correction, Hispanics and African Americans scored significantly higher on the distress thermometer than Caucasians**
Chen et al. (2020) ^a^	USA, multi-sites including three paediatric hospitals and two adult hospitals	Longitudinal cohort study 4 time points: T0 = within 4 months of diagnosis, T1 = 6 months, T2 = 12 months, T3 = 24 months	*n* = 179 (46.9) Age = 15–39 23.41 (9.09) Numerous and heterogeneous cancer types, stages and treatment status	White (78) Hispanic/Latino (78) Other (21) Missing (2)	BSI-18 PDS PTGI SF-36	**AYAs with high-distress profiles were less likely to be Hispanic/Latino than White, relative to those with resilient-growth profiles**
DeRouen et al. (2015)	USA, population-based	Retrospective, cross-sectional study	*n* = 484 (36) Age = 18-39 Mean NR, 95% of participants were aged 20 or above. Numerous and heterogeneous cancer types, stages and treatment status	Non-Hispanic White (284) Hispanic (102) Non-Hispanic Black/American Indian/Alaska Native (50) Non-Hispanic Asian/Pacific Islander (48)	SF-12	No statistically significant differences were found in the mental component QoL scores between Hispanic, Black, American Indian, Alaska Native, Asian and Pacific Islander and White AYAs
Desai et al. (2021)	USA, population-based	Cross-sectional study	*n* = 1025 (100) Age = 18–40 33.3 (4.9) Numerous and heterogeneous cancer types, stages and treatment status	White (745) African American (29) Asian or Native American (74) Mixed/other (177)	PHQ-8 GAD-7	There was no statistically significant association between depression, anxiety scores and different ethnic groups
Husson et al. (2017) ^a^	USA, multi-sites including three paediatric hospitals and two adult hospitals	Prospective, longitudinal cohort study 3 time points: T1 = within 4 months post-diagnosis, T2 = 12 months, T3 = 24 months	*n* = 176 (44.9) Age = 15–39 23.6 (8.9) Numerous and heterogeneous cancer types, stages and treatment status	White (81) Hispanic/Latino (73) Other (20)	SF-36	No significant differences in the emotional component of QoL scores were found between ethnic groups at all time points
Kwak et al. (2013b) ^a^	USA, multi-sites including three paediatric hospitals and two adult hospitals	Prospective, longitudinal cohort study 3 time points: T1 = within 4 months post-diagnosis, T2 = 6 months, T3 = 12 months	*n* = 215 (47) Age = 14–39 23.6 (8.9) Numerous and heterogeneous cancer types, stages and treatment status	White (95) Hispanic/Latino (91) Other (27)	BSI-18	**Hispanic/Latino or other minoritised participants reported significantly higher mean global symptom index scores than White AYAs at baseline**
King-Dowling et al. (2023)	USA, multi-sites including three hospitals	Cross-sectional study	*n* = 531 (52) Age = 15–29 19.41 (2.6) Numerous and heterogeneous cancer types, stages and treatment status	Non-Hispanic White (347) Black, Indigenous, Hispanic and people of Colour (181)	CEFIS-AYA	**Black, Indigenous, Hispanic and people of Colour had significantly higher distress scores than non-Hispanic Whites**
Munoz et al. (2016)	USA, hospital-based	Cross-sectional mixed methods study	*n* = 31 (64.5) Age = 18–39 33.2 (5.1) Numerous and heterogeneous cancer types, stages and treatment status	Hispanic (13) Non-Hispanic Black (9) Asian/Pacific Islanders (8) Mixed (3) Other (5)	FACT-G	**Asian/Pacific Islanders reported a significantly better emotional QoL score than Hispanics.** **Asian/Pacific Islanders also reported the best overall QoL scores, followed by Black, non-Hispanics and Hispanics**
Ritt-Olsen et al. (2018)	USA, multi-sites including two hospitals	Cross-sectional study	*n* = 194 (49.36) Age = 15–25 20.75 (2.77) Numerous and heterogeneous cancer types, stages and treatment status	Latino (91) Non-Latino: Including African American, White, Asian (103)	CES-D PedsQL	**Latinos experienced significantly higher depressive symptom scores of clinical significance and lower QoL scores than AYAs identified as non-Latinos**
Smith et al. (2013)	USA, population-based	Cross-sectional study	*n* = 523 (33.08) Age = 15-39 Mean NR Numerous and heterogeneous cancer types, stages and treatment status	White (285) Hispanic (100) Black (43) Other (56)	SF-12 PedsQL	There was no significant difference in the mental component of SF-12 QoL scores between Hispanic, Black and White AYAs
Zebrack et al. (2014)	USA, multi-sites including three medical institutions	Prospective, longitudinal cohort study 3 time points: T1 = within 4 months post-diagnosis, T2 = 6 months, T3 = 12 months	*n* = 215 (46.98) Age = 15–39 23.6 (8.9) Numerous and heterogeneous cancer types, stages and treatment status	Non-Hispanic White/Caucasian (95) Hispanic/Latino (91) African American (11) Asian/Pacific Islander (13) American Indian/Alaska Native (3)	BSI-18	There was no significant association between ethnic groups and assignments to the four distress trajectory groups (chronic distress, Delayed, Recovery and Resilient). The odds ratio of White versus non-White AYAs in predicting the likelihood of being categorised in the resilient group was also insignificant
Zebrack et al. (2015) ^a^	USA, multi-sites including three paediatric hospitals and two adult hospitals	Prospective, longitudinal cohort study 3 time points: T1 = within 4 months post-diagnosis, T2 = 6 months, T3 = 12 months	*n* = 165 (46.1) Age = 14–39 22.8 (8.8) Numerous and heterogeneous cancer types, stages and treatment status	White/Caucasian (74) Hispanic/Latino (71) Other (18)	PTGI PDS	No significant differences in post-traumatic stress severity scores were found between White and non-White participants. **Non-White AYAs had a significantly higher score in the new possibilities domain of PTGI.**

^a^indicates the studies with suspected duplicate datasets, **Bold** highlights findings that reported significant differences between ethnic groups.

**Abbreviations**: SD, standard deviation; BSI-18, Brief Symptom Inventory-18; PDS, Posttraumatic stress diagnostic scale; PTGI, Posttraumatic Growth Inventory; SF-36, Medical Outcomes Study Short Form-36 Health Survey; NR, not reported; SF-12, Short Form Health Survey; QoL, Quality of Life; PHQ-8, Patient Health Questionnaire-8; GAD-7, Generalised Anxiety Disorder Assessment-7; CEFIS-AYA, COVID-19 Exposure and Family Impact Scales for Adolescents and Young Adults; FACT-G, Functional Assessment of Cancer Therapy-General; Centre for Epidemiologic Studies Depression Scale; PedsQL Scale, Paediatric Quality of Life Inventory.

### Quality appraisal

As all studies utilised an observational design, there are constraints to the extent of methodological rigour that can be achieved when compared to randomised controlled trials. Five studies were longitudinal with reasonable follow-up periods spanning from 12 to 24 months, though four were suspected to have overlapping datasets. Seven studies adopted a cross-sectional design. Overall, longitudinal studies had higher ratings: only Kwak et al. (2018b) was rated as strong, whilst eight studies were rated as moderate and three had low ratings (supplementary file 2). The main methodological concerns were self-reported outcome measures that failed to address confounds and were only partially validated for some participants. Additionally, ethnic groups were poorly classified with wide variations across studies and some races were clustered together in the analysis as one ‘non-White’ group. The discrepancy in categorization made it challenging to draw meaningful comparisons between groups and neglected the diversity in psychological outcomes amongst minoritised ethnicities.

### Psychological outcomes

Disparities in psychological outcomes between ethnic groups were demonstrated in six studies. The majority highlighted that ethnically minoritised AYAs with cancer experienced poorer psychological health compared to Caucasian AYAs. Burgoyne et al. (2015) only provided data on the mean and standard error of distress thermometer score by ethnicity. The differences in means between groups were hand computed using the Independent Samples *t* test, followed by a Bonferroni correction to counteract issues arising from multiple comparisons. The level of significance after the Bonferroni adjustment for multiple comparisons was *p = .0125.* In the analysis between Caucasian and Hispanic AYAs, the *t* test revealed a significant difference in scores, t (532) = 3.18, *p* < .01, with Hispanics reporting significantly higher distress scores than Caucasians. The effect size, as indicated by Hedges’ g due to unequal sample size, was moderate with a value of 0.59. Similarly, the comparison between Caucasians and African Americans showed t (602) = 2.63, *p* < .01, suggesting that significantly more distress was reported by African Americans, with a small Hedges’ g effect size of 0.29. No significant difference was found between Hispanics and African Americans. Kwak et al. (2013a) and King-Dowling et al. (2023) both combined minoritised groups into one and compared outcomes with White AYAs. The former found that minoritised participants reported a significantly higher mean Global Severity Index (GSI) score as derived from their depression, anxiety and somatization subscales. This is corroborated by the latter researchers, who showed that People of Colour as a whole had a higher COVID-19 related distress score than White AYAs.

Munoz et al. (2016) conducted a more in-depth comparison between individual minoritised groups. They illustrated that Asian/Pacific Islanders reported significantly better emotional well-being scores than Hispanics. This group also described the best overall QoL score, followed by Black, non-Hispanic and Hispanic AYAs. This study lent support to Burgoyne et al.’s (2015) findings that underscored the psychological health of Hispanic AYAs seemed to fare worse than other minoritised AYAs, whilst Asian/Pacific Islanders do better.

Similarly, Ritt-Olsen et al. (2018) investigated depressive symptoms and QoL between Latino and non-Latino AYAs, including African Americans, Caucasians and Asians as one group. Additionally, they examined the role of acculturation by degree of orientation to the Anglo or Mexican/Latino culture in association with these outcome measures. They demonstrated that Latinos experienced significantly higher levels of depressive symptoms that exceeded clinical threshold and described lower QoL scores compared to non-Latino AYAs. Furthermore, cultural analysis revealed that more acculturated Latinos (lower Latino and higher Anglo culture orientation) had higher depressive scores reaching a clinical threshold. A similar trend was observed for the QoL measure, where poorer QoL were described by more acculturated Latinos.

By contrast, only one study showed that ethnically minoritised groups reported better outcomes. Chen et al. (2020) classified respondents’ profiles based on a combination of distress scores informed by BSI-18, PDS and PTGI: distressed (high distress/low growth), distressed growth (high distress/high growth), resilient (low distress/low growth) and resilient growth (low distress/high growth). They found that young people with high distress profiles were more likely to be White than Hispanic/Latino, in relation to those with resilient growth profiles. The remaining six studies found no significant differences or odds ratios in psychological outcomes between ethnic groups (DeRouen et al., 2015; Desai et al., 2021; Husson et al., 2017; Smith et al., 2013; Zebrack et al., 2014; Zebrack et al., 2015).

#### Longitudinal outcomes

Kwak et al. (2013a) analysed the change in distress trajectory by ethnicity from receiving a diagnosis to one year post-diagnosis. They demonstrated that minoritised AYAs, despite having a higher GSI score at baseline, experienced a significant linear decline in their GSI score over time. However, White respondents, although reported a significantly lower baseline GSI score, their levels of distress did not reduce markedly over one year.

Likewise in Husson et al.’s (2017) study, although there was no significant difference in the baseline measures amongst ethnic groups initially, within-group comparisons over time showed that only Latino AYAs reported a significant improvement in psychological QoL scores over 24 months. This result mirrored Kwak et al. (2013b)’s observation that psychological outcomes in minoritised AYAs appeared to ameliorate considerably compared to their White counterparts regardless of their baseline distress levels.

## Discussion

The present review explored psychological outcomes in ethnically minoritised young cancer patients. Despite some mixed findings, five studies demonstrated that minoritised AYAs reported significantly higher distress levels than White AYAs, which appeared to mirror the Minority Stress Model (Meyer, 2003). The convergence of multiple minority stressors is associated with compounded challenging experiences (Shangani et al., 2020). The racial discrimination minoritised AYAs might face compounded on the stress and social isolation from cancer could contribute to poorer psychological outcomes. Additionally, adjustment to the dominant culture was associated with higher levels of psychological distress (Da Silva et al., 2017). These factors underline the impact of minority group status on observed disparities in psychological wellbeing.

Amongst the minoritised groups, Hispanic AYAs consistently showed poorer psychological outcomes compared to other minorities. This finding supported Luckett et al.’s (2011) conclusion that disparities between majority and minoritised patients were driven largely by those from a Hispanic ethnic origin. Communication barriers even in the presence of interpreters, financial restraints, negative perception of care were recurrent themes associated with poorer health outcomes in Hispanics (Mayo et al., 2007). The impact of acculturation on mental health outcomes of Hispanic youths has also garnered attention in the literature and resembles Ritt-Olsen et al.’s (2018) findings in this review. Acculturation is a risk factor for depression as higher acculturation into the dominant culture was associated with increased depressive symptoms (Lorenzo-Blanco et al., 2012). Post-traumatic growth was also found to be significantly lower in acculturated Hispanic childhood cancer survivors who spoke English as their first language (Arpawong et al., 2013). Hispanic cultural values emphasize family cohesion and a strong sense of community (Rivera et al., 2008), which may mitigate the effects of psychological distress. Acculturation, however, is hypothesized to contribute to deterioration in family functioning and closeness as Hispanic values become diluted (Sullivan et al., 2007). Cultural identification intersects with the Minority Stress Model that implies although minoritised groups are confronted with discriminatory challenges, their cultural values could serve as a protective buffer against these stressors and strengthen resilience.

Nonetheless, the inconclusive findings in this review suggest that there could be other mediating factors influencing psychological outcomes. Minoritised groups tend to be a proxy measure for socioeconomic disadvantage, which has direct implications to inequalities in accessing healthcare and insurance. (Ng et al., 2019). Low income and unemployment were associated with diagnostic delays and poorer mental health in AYA cancer patients (Tanner et al., 2023). The lack of financial stability and insurance coverage can influence the quality and continuity of care, exacerbating worries and distress (Salsman & Kircher, 2022). Furthermore, the cross-sectional design in seven studies recruited participants at various stages of cancer and treatment. Patients with certain cancer types, who underwent surgery have more favourable psychological outcomes compared to those receiving chemotherapy and radiotherapy (Tanner et al., 2023). Therefore, the mixed findings could be an artefact of cross-sectional designs that failed to account for cancer-related factors which could impact negatively on AYAs’ mental health. This underlines the value of longitudinal research in elucidating the dynamic interplay between personal, cancer-related factors and psychological outcomes over time.

### Strengths and limitations

The present review is the first to date that systematically evaluates the literature on psychological outcomes in young ethnically minoritised cancer patients. In light of the global priority in addressing inequalities in cancer care (The Lancet Oncology, 2021), this review contributes to the evidence base by highlighting ethnic disparities that stem early from adolescence. The review is also strengthened by its high transparency and minimisation of bias by adhering to the PRISMA statement and registration on PROSPERO.

Nevertheless, the findings of this review should be considered in light of several limitations. One important limitation is that all twelve identified studies were conducted in the US, where healthcare is largely privatised and hence introduces biases related to financial disadvantage that could impact on access to healthcare services and subsequently, health outcomes. This substantially limits generalisability as the papers failed to capture the vast diversity across ethnic groups and healthcare services internationally. Another limitation is the unaddressed confounds in several studies that weakens the validity of findings as the observed differences may be attributable to confounding factors other than ethnic differences. The inconsistent and flawed reporting of ethnic groups alongside the persistent small sample sizes in minoritised groups have also posed difficulties in drawing comparisons and interpreting inter-ethnic differences. Moreover, the exclusion of participants experiencing high distress and who could not read English may have failed to capture the full spectrum of AYAs with distress, underestimating the true prevalence of psychological distress amongst minoritised AYAs with cancer.

### Clinical implications

Findings implied that ethnically minoritised AYAs with cancer are at risk of experiencing poorer psychological health compared to their White peers, underlining the importance of psychosocial support alongside medical treatments. Longitudinal studies revealed that psychological well-being tends to be at its worst following diagnosis, then fluctuate around six to twelve months and gradually improve over time. Comprehensive psychosocial assessment should be routinely evaluated at critical points throughout AYAs’ cancer journey, for instance at diagnosis and at regular intervals thereafter to ensure AYAs’ changing needs and engagement over time are accommodated. Professionals should receive routine training in enhancing cultural competence and sensitivity whilst providing care. Promoting clear and effective communication, interpersonal control, and showing warmth and respect have been underlined as fundamental elements of culturally sensitive care, which predicted improved health outcomes, treatment adherence and satisfaction (Tucker et al., 2011).

Moreover, findings from the US have highlighted Hispanic/Latino AYAs as most at risk of experiencing poorer mental health. Yet, in the UK where there is currently no ethnic category for Hispanic/Latino individuals recorded in the government or hospital databases, little is known about their experiences in the UK. This raises questions as to the extent to which difficulties that Hispanic youths face are culture bound to the systemic prejudice towards Latin Americans within the US, or whether this represents a broader phenomenon amongst Hispanic migrants living in other Western countries. The added complexity of intersectionality, where different forms of social factors, such as poverty and migration, compound themselves and contribute to marginalisation should be taken into consideration in understanding AYAs’ experiences and informing support plans. Clinicians should attend to minoritised AYAs’ ethnic identity and avoid categorising them to the ‘other’ group, overlooking the associated difficulties they may face. Clinicians could also explore AYAs’ cultural orientation and their extent of acculturation, which may be useful in understanding the impact of cultural influences underlying health behaviours and outcomes.

### Future research

Despite emerging literature highlighting ethnic disparities in cancer, qualitative research focusing on the experiences and needs of minoritised AYAs is scant. There needs to be amplified efforts in recruiting ethnically minoritised young people in research to enhance visibility and representativeness. Attention must be given to the way ethnicity data are recorded to avoid reducing the heterogeneity of ethnic groups. For example adopting standardized ethnicity classification aligning with the national census, and avoiding overly broad groupings, such as ‘non-White’, to improve the accuracy and applicability of findings. Future research could benefit from examining broader psychological domains as some only measured the emotional component of QoL, for example trauma, obsessive-compulsive disorder as well as positive outcomes: self-esteem, resilience and post-traumatic growth. The use of clinical interview could also overcome biases in self-reported questionnaires and facilitate a more culturally sensitive approach to accommodate individuals from diverse ethnic backgrounds.

Further longitudinal data could delineate the changes in psychological outcomes over time from diagnosis into survivorship. Understanding the trajectory allows clinicians to tailor more personalised care at different stages of their cancer journey to meet AYAs’ evolving needs. Additionally, whilst AYAs oncology research focuses on ages 15 to 39 to recognise their unique psychosocial needs, the vast heterogeneity within 24 years range complicates generalisability and overlooks the idiosyncratic experiences of individuals across the spectrum. Further breakdown of age groups is required to develop a more comprehensive understanding of how different age cohorts are impacted psychologically at specific life stages.

## Conclusion

The review extended the work of Luckett et al. (2011) in highlighting ethnic disparities in psychological outcomes amongst young cancer patients. Although findings were mixed, there was evidence suggesting minoritised AYAs, especially Hispanics/Latinos, reported higher levels of distress compared to their White peers. However, these findings are limited by methodological issues that weakened their validity. Further cross-cultural research is warranted to illuminate factors contributing to these ethnic disparities and elucidate issues relating to the intersectionality of culture, social determinants, and structural racism.

## Supplemental Material

Supplemental Material - Psychological outcomes in ethnically minoritised adolescents and young adults with cancer: A systematic review Supplemental Material for Psychological outcomes in ethnically minoritised adolescents and young adults with cancer: A systematic review by Lynette WS Chan, Alan Hebben-Wadey, Chandrika Kambakara Gedara and James McParland in Clinical Child Psychology and Psychiatry
